# Approaches for the preparation and evaluation of hydrophilic polyethylene and polyethylene terephthalate microplastic particles suited for toxicological effect studies

**DOI:** 10.1007/s00216-024-05726-7

**Published:** 2025-01-25

**Authors:** John Seghers, Claudia Cella, Emmy Pequeur, Rita La Spina, Francesco Roncari, Andrea Valsesia, Dora Mehn, Douglas Gilliland, Stefanie Trapmann, Håkan Emteborg

**Affiliations:** 1 European Commission, Joint Research Centre (JRC), Geel, Belgium; 2 European Commission, Joint Research Centre (JRC), Ispra, Italy; 3 Laboratory of Environmental Toxicology and Aquatic Ecology, Faculty of Bioscience Engineering, (GhEnToxlab), Ghent University, Ghent, Belgium

**Keywords:** Microplastic, Surfactants, Bio-availability, Hydrophilicity, Artificial ageing, Toxicity, Effect studies, Qualitative reference materials, Ordinal property, Hydrophobicity index

## Abstract

When performing effect studies to investigate the impact of microplastic (MP) on cell lines, algae, or daphnia, it is advantageous if such experiments can be performed without the use of surfactants. The need for surfactants arises from the fact that finely milled pristine MP particles generally are hydrophobic. Methods for the preparation of larger amounts of hydrophilic and hence artificially aged MP particles and approaches for their characterization are of high importance. Here we present methods to artificially age polyethylene terephthalate (PET) and low-density polyethylene (PE) using alkaline and acidic treatments that reproducibly result in large quantities of particles below 5 µm with considerably increased hydrophilicity. The artificially aged MP particles were characterized using particle counting by single-particle extinction and scattering (SPES), particle size by laser diffraction measurements, zeta potential using electrophoretic light scattering, hydrophobicity index (Hy) through dark-field (DF) microscopy, chemical composition by inductively coupled plasma-mass spectrometry (ICP-MS), Fourier transform infrared (FTIR) microscopy, and Raman microscopy. The hydrophobicity index values obtained should allow the aged MP particles to be characterized as qualitative reference materials (RMs) with an ordinal property. Evidence for the maintained integrity and hydrophilicity of the artificially aged MP particles (in powder form) over time was obtained by measurements of zeta potential with a 33-month interval. Uniformity of subsampling with respect to particle number concentration in suspensions within a 10-day period was also investigated. It provided evidence for the possibility of reproducible spiking of a specific number of hydrophilic MP particles with relative standard deviations (RSDs) from 6.2 to 13.6%. For the development of future reference materials of artificially aged microplastics, they should be characterized for an ordinal property (artificial age as Hy-index) and nominal property (identity of PET or PE based on spectral matching).

**Graphical Abstract:**

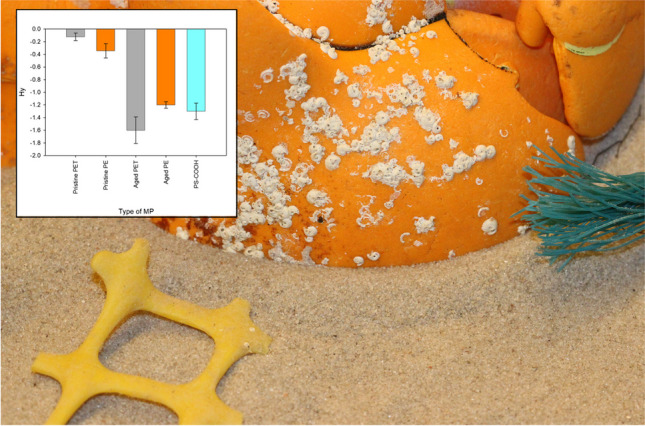

**Supplementary Information:**

The online version contains supplementary material available at 10.1007/s00216-024-05726-7.

## Introduction

Most microplastic (MP) fragments (< 5 mm in size) found in the environment originate from larger plastic objects [1]. These secondary MP particles are the result of mechanical degradation, photo-chemical weathering, and oxidation processes of the larger plastic items that gradually result in a myriad of smaller fragments with increasing hydrophilicity [2–4]. Notably, some literature states that nanoplastics are particles with diameters < 1 µm [5, 6]. For actual nanomaterials as defined in the European Commission (EC) Recommendation, the cut-off is set at 0.1 µm [7]. Among the artificially aged MP particles prepared in this work, there are initially fractions that can be considered nanoplastics although mainly present *prior* to the filtration step using a 0.2 µm filter. The remaining text therefore uses the word microplastic, even though smaller particles are still present as minute particles can adhere to the surface of larger particles.

Natural weathering processes reduce the size and modify the particles’ surfaces by changing structures thereby introducing (or exposing) polar groups with a gradual fragmentation and coverings with bio-film [8]. Fotopoulou et al. assessed the properties of beached plastics pellets in comparison with pristine pellets. For PE, they found eroded surfaces and negatively charged functional groups on the surface that rendered the pellets a negative surface charge at seawater pH [9]. Such processes make the aged particles much more hydrophilic than in their pristine state. Due to the relative inertness of plastic objects, these natural processes take substantial time as smaller fragments are subsequently peeled off from oxidized surfaces from larger objects. Eventually, the wettability and bio-availability of the resulting MP particles increase [2–4, 8, 9]. Because of these processes, the naturally weathered MP particles have differing properties in comparison with pristine MP fragments of unaged polymers especially with respect to hydrophilicity.

Alimi et al. recently reported that more than 90% of the micro- and nanoplastic used in effect studies were still made up of pristine plastic particles [3]. These authors furthermore concluded that understanding the true risks associated with MP pollution may be limited because of the physiochemical differences between pristine and naturally weathered MPs. In addition, among the 10% of studies using aged MPs, polystyrene was the most studied polymer whereas polyethylene is by far the most common polymer in environmental samples. Hence, access to artificially aged PE (preferably in the form of reference materials) is particularly relevant for future effect studies. A recent stakeholder survey on MP needs (JRC137948) shows the high need/demand for (artificially) aged MP particles of smaller size classes very clearly [10]. When investigating the current availability of artificially aged MP RMs, only one reference material appears to be available, namely BAM-P201, which stems from a UV-aged PE film [11]. The intended use of the BAM material is one-shot, i.e., use of the entire contents (10 mg) and it has particle sizes up to 300 µm [11]. Moreover, other approaches for the preparation of MP RMs have been published and were recently made commercially available [12–14]. In the latter reports, however, the MP RMs are made of pristine particles and are not artificially aged or hydrophilic [12–14]. A consolidated approach for the preparation and supply of hydrophilic MP particles that fulfil the RM definition is undoubtedly important. This work outlines such approaches combined with measurements of their hydrophobicity index. The hydrophilic MP particles should be modified in such a way that they behave as (artificially) aged MP in subsequent experiments. This study is the first of its kind to describe Hy-index measurements for the assessment of hydrophilic/artificially aged MP particles.

In contrast to naturally aged MP, pristine MP particles are hydrophobic. Therefore, surfactants such as Triton-X 100 are used to make them hydrophilic enough to suspend them into an aqueous suspension which is the medium of choice for effect studies as reported by Chen et al. [15]. In the absence of surfactants, such pristine polymers (PE and polypropylene (PP) remain on the liquid surface in static experiments because of their density and hydrophobicity. Exposure experiments to cells, algae, or small organisms such as daphnia become unrealistic in comparison with naturally aged MP particles because of the way the pristine particles are suspended and evidently also because naturally aged MP particles are hydrophilic [2–4, 8, 9].

The toxicity and risks of different surfactants and their degradation products in environmental matrices and effects on cell lines and other bio-markers are at the same time well-known [15–18]. However, it is difficult to identify which agent (the surfactant, the MP particle, or even a combination of both) has caused the observed effect(s) on exposed organisms and cell lines if surfactant-stabilized MP is used.

Evidently, one could sample naturally aged secondary MP particles and use these for effect studies. However, these secondary MP particles are not the result of controlled degradation processes but originate from random ageing and fragmentation processes and therefore make up heterogeneous mixtures of particles with respect to origin and composition [19]. Effect studies using naturally weathered MP might therefore suffer from the risk of lower reproducibility since any new batch of aged MPs sampled from the environment will be somewhat different. If one would use natural processes for degradation, it would also take substantial time (years) for plastic present in a (semi-controlled) environment to age sufficiently [2].

Consequently, there is a higher possibility of equivalence between different batches of artificially aged MP particles compared to naturally weathered MP. Liu et al. compiled data from numerous studies of artificial ageing of MP [19]. They found that about 65% of the articles had employed UV exposure to achieve ageing whereas chemical oxidation was used in 18% of the studies. Liu et al. also listed a number of potential differences between naturally weathered plastics and artificially aged MP and they pointed out that artificial ageing processes are less complex than MP particles aged in the environment. Artificially aged MP prepared as described in this work can be obtained much faster and in higher quantities by using more aggressive agents (acid and base) than the natural oxidative processes. This is achieved by treating cryo-milled particles that are already substantially reduced in size. The artificially aged PET particles prepared using the schemes described here were used in toxicological effect studies described elsewhere [20–22].

Von der Esch et al. used an alkaline hydrolysis method to degrade PET by applying potassium hydroxide (KOH) in combination with ultrasonication to produce aged particles from larger plastic pieces [23]. The final number of particles obtained from a 1 cm^2^ square of PET polymer was rather limited and ranged from 17,000 to 71,000 particles from 1 μm to 1 mm. The low material output from ageing of larger objects in climate chambers or ultrasonic baths is a clear drawback. The very low amount supplied (10 mg) for BAM-P201 also suggests a limitation in the amount of aged bulk material obtained from the ageing process [11]. In this work, we employed mechanical and chemical treatments on already cryo-milled fragments so that more than 10 million artificially aged particles (< 5 µm) per milliliter of suspension could easily be obtained after dilution (the number of particles/milliliter required can be adapted using different dilution factors). For PET, this was achieved by directly treating the smaller cryo-milled PET fragments in an alkaline suspension. To artificially age cryo-milled PE, we oxidized the polymer surface with strong oxidizing agents like HNO_3_ and H_2_O_2_ at an elevated temperature.

Such MP materials could become reference materials characterized for qualitative properties. As outlined in ISO 33406, qualitative properties can be nominal or ordinal [24, 25]. In contrast to quantitative properties, qualitative properties only allow comparisons. Comparison of nominal properties only allows to conclude that they are identical or different. Comparison between ordinal properties are of relative order and allow to state whether one is lower, equal to, or higher than the other [24, 25]. One assigned property for the MP materials discussed here could be the identity of the material, e.g., PET, a nominal property, and the second assigned property of ordinal nature, indicating the artificial ageing process (“less aged,” “aged,” “more aged”), confirmed by increasing hydrophobicity indices (Hy-index). The nominal property, the identity of the polymers, can be confirmed by various methods (e.g., IR and Raman spectra), while the ordinal property can be assigned relative to the exposure time, reagent concentration, and temperature used for an artificial ageing process (i.e., an operationally defined chemical treatment making the materials hydrophilic) [24, 25].

Preliminary categorization and ordinal ordering of the artificially aged MP particles was confirmed using measurements of Hy-index. The Hy-index which is described in more detail under a separate sub-heading is a measurement of affinity of particles towards a hydrophobic surface [26–30].

As with all reference materials, assessment of homogeneity and stability is mandatory also for qualitative RMs [31]. In this work, we assessed the stability of hydrophilicity of the artificially aged MP particles by measuring zeta potential in aqueous suspensions. Hildebrandt et al. also used zeta potential to characterize polypropylene particles of about 0.2 µm size that had been prepared using an Ultra-Turrax disperser at 18,000 rpm [32]. The zeta-potential in suspensions prepared from artificially aged PET and PE (stored as powders) was measured with a 33-month interval. It was also important to assess the variation of the specific number of MP particles per milliliter from repeated subsampling with the notion that the “age” e.g. hydrophilicity is unchanged. Therefore, we investigated the number of particles present in 0.5 ml portions from suspensions prepared from two different preparations of aged MP of PE and PET which was a study of uniformity of the prepared suspensions. The “homogeneity of ageing” based on Hy-index measurements was not assessed during these studies.

For molecules and particles that are small enough, a high zeta potential will confer stability, i.e., the solution or suspension will resist aggregation. Colloids with high zeta potential (negative or positive) are hence stabilized [33, 34]. In this work, the zeta potential value (in mV) was evaluated for the artificially aged MP particles. In this sense, the zeta potential showing the stability of the suspensions is a proxy for assessing the artificial age of the treated MP particles. Unfortunately, it is difficult to measure the zeta potential of hydrophobic pristine MP fragments due to the need for a uniform suspension during the measurement. Thus a measurement of zeta potential is only possible (in this case) if the particles can be suspended in a conductive medium such as a 1 mM phosphate buffer whereby no direct comparison of zeta potential can be made between hydrophilic and hydrophobic MP particles.

This work describes methods for chemical treatment of MP particles of PET and PE that have been characterized using a multitude of analytical techniques. Since already milled MP particles are treated, large quantities of artificially aged MP can be obtained within a couple of days allowing every surface to become hydrophilic. The main use of artificially aged MP particles is for toxicological effect studies as they more closely mimic naturally aged MP particles in comparison with pristine MP particles. The artificially aged MP particles were characterized using Hy-index [26–30].

## Experimental

### Preparation of aged PET

Here, 5 g of PET powder (Goodfellow, Cambridge, UK, Lot No. 300830480) of nominally 300 µm was cryogenically milled 60 times 3 min in a stainless steel milling chamber with intermediate cooling intervals of 3 min using liquid nitrogen in a SPEX SamplePrep Freezer-Mill 6870 (Cole Parmer, Vernon Hills, USA). The technology uses a dual electromagnetic, grinding chamber that rapidly drives a steel impactor back and forth against the two end plugs of steel in the sample holder. The milling chamber is immersed in liquid nitrogen at − 196 °C throughout the grinding process.

Out of the initial 5 g Goodfellow PET, 4.78 g of finely milled PET powder was recovered from the chamber after milling. All subsequent work was performed in a clean bench NUAIRE/FUMEGARD, NU-156-524E (Plymouth, MN, USA). All equipment used had been sonicated and cleaned with ultrapure Type-1 water (resistivity of > 18 MΩ-cm^−1^, a conductivity of < 0.056 µS/cm, and < 50 µg/l of total organic carbon (TOC)) and ethanol to minimize contamination of the samples [35]. A 50 ml 1 M KOH solution (VWR) was similarly prepared in Type-1 water and filtered over a 0.2 µm cyclopore polycarbonate track-etched membrane (Whatman, Maidstone, UK) to remove foreign particles. Next, 50 ml 1 M KOH solution was transferred to a 250-ml glass beaker, and 4.5 g of the cryogenically milled PET was added and it was subsequently etched in two steps. The resulting suspension was mixed for 15 min using a glass-coated magnetic stirring bar. Thereafter, the magnetic stirring bar was removed and 150 ml of Type-1 water was added to dilute the KOH concentration to 0.25 M. The glass beaker was then placed in an ultrasonic bath and sonicated at 42 kHz 100 W (Branson 2510E-MT, St. Louis, MO, USA). Crushed ice was added to the water bath to keep the temperature under control and excess water was removed to the keep water level at maximum fill height. Every 15 min, the mixture was stirred with a stainless steel spoon and the total reaction-sonication time was 5 h and 30 min. The suspension was then vacuum-filtered over a 47 mm diameter, 0.2 µm cyclopore polycarbonate black track-etched filter (Whatman, Maidstone, UK). The resulting particles were washed six times with 50-ml portions of Type-1 water to remove excess KOH. The pH was checked with a test strip to confirm neutral pH before proceeding. The filter holder, filter, and PET cake was finally placed in a drying oven NAPCO, model 5831 (Thermo-Fisher, Marietta, OH, USA), at 60 °C for 24 h. After drying, 4.15 g of dried aged PET powder was recovered. A flow diagram of the preparation of aged PET is presented in the supplementary material (Figure S1). The resulting aged PET particles were then stored as a dry powder kept at room temperature until further use.

### Preparation of aged PE

Initially, 5 g of Alfa Aesar LDPE flakes of about 2 to 6 mm length was placed in a 304 stainless steel milling chamber. These flakes had been cut with a knife from a 30 cm × 6.25 mm diameter LDPE rod (Alfa Aesar, Thermo Scientific, Brussels, BE). The steel chamber was subsequently immersed in liquid nitrogen contained in a cryogenic mill (SPEX SamplePrep Freezer-Mill 6870, Cole Parmer, Vernon Hills, USA). In the case of PE, intermediate cooling intervals of 4 min were used. The resulting powder in the milling chamber was recovered and 4.638 g of LDPE powder was available after the milling step. All subsequent manipulations were performed in a clean bench (NUAIRE/FUMEGARD, NU-156-524E) and all equipment was sonicated and cleaned with Type-1 water and ethanol to minimize contamination of the samples. All solutions employed were first filtered over 0.2 µm to remove foreign particles using cyclopore polycarbonate track-etched membrane (Whatman, Maidstone, UK).

Thereafter, 0.5024 g of cryo-milled Alfa Aesar PE was placed in a beaker with 20 ml HNO_3_ (Merck, Darmstadt, DE; EMSURE Nitric Acid 65%) and 5 ml 30% H_2_O_2_ (Merck, SUPRAPUR Hydrogen Peroxide 30%, Merck, VWR, BE). Subsequently, the mixture was heated to 70 °C for 30 min, while stirring with a glass-coated magnetic stirring bar. At this point, the suspension turned brown due to the decomposition of the HNO_3_. After 30 min, the HNO_3_/H_2_O_2_/LDPE suspension was filtered over a black 47 mm 0.2 µm polycarbonate track-etched filter (Whatman). The PE particles were captured on the filter surface using a glass funnel rinsed with 50 ml Type-1 water. On the filter surface, the PE particles were then rinsed with 4 times 50 ml of 0.1 M KOH and rinsed with 4 times 50 ml of Type-1 water to remove any remaining HNO_3_, KNO_3_, or KOH solution. This was done to neutralize the remaining HNO_3_ and to convert the COOH groups on the polymer surface to COOK. The filter set-up holding the aged LDPE was placed at 60 °C for 24 h to dry. The dried particles were removed from the filter collecting 0.486 g of dried and artificially aged LDPE. A flow diagram of the preparation of aged PE is presented in the supplementary material (Figure S2). The resulting aged PE particles were then stored as a dry powder and kept at room temperature until further use.

### Measurements on aged PET and PE particles using an array of techniques

#### Measurement of zeta potential of PET using electrophoretic light scattering—related to stability of suspensions

A PET suspension was prepared by placing 1.4 g of dried aged PET in 50 ml Type-1 water by sonication and stirring (15 min). The suspension was pulse-filtrated (push/release/retract/push…) over a 5 µm syringe inline filter (PALL, Acrodisc 32 mm Syringe Filter, 5 µm Supor Membrane). The zeta potential measurements were subsequently performed on 20 ml of filtrate using a Zetasizer ZS instrument (Malvern Panalytical, Malvern UK) in 1 mM phosphate buffer (pH 7) using a clear disposable zeta cell. Six replicate measurements of zeta potential were performed where two subsamples were measured in triplicate. The sample was introduced by means of a peristaltic pump at 4 ml/min. One measurement sequence lasted about 2 min.

#### Measurement of zeta potential of PE using electrophoretic light scattering—related to stability of suspensions

The PE particles were similarly suspended in a 1 mM phosphate buffer (pH 7) as described above and measured using the Zetasizer ZS instrument (Malvern Panalytical, Malvern, UK).

#### Hydrophobicity index, Hy

The hydrophobicity index was determined following the method outlined in the OECD Test Guideline 126 [26]. This method was first introduced for assessing various nanomaterials and nanoparticles by Desmet et al. [27], Desmet et al. [28], Valsesia et al. [29], and Roncari et al. [30]. The Hy-index of the artificially aged MP was obtained after filtration through a cellulose acetate filter with a cut-off value of < 1.2 µm. Dark-field microscopy was employed to capture image sequences of 25 frames for a duration of 12 min. These image sequences were then used to quantify the displacement of the MP particles by measuring the scattered light from them on the surface of the hydrophobic collector surface. A volume of 20 µl was injected with a pipette in a 16-channel microfluidic chamber (Chipshop, Jena, DE) filling each channel completely. The number of particles binding to the collector per frame was monitored using the software ImageJ and its Trackmate plugin. Particle detection was performed by thresholding the pixel intensity distribution from the dark-field microscopy images. The analysis consists in an automatic detection of the particles per frame, and the tracking of their positions within the sequence of frames. This automatic calculation quantifies the number of bound particles per frame. The actual time elapsed was calculated by multiplying the frame number by the frame delay (30 s), to obtain the number of bound particles per unit of time which corresponds to the binding rate of the hydrophilic MP material onto the collector. The binding rates were then used to calculate the hydrophobicity index Hy, defined as the logarithm of the ratio between the binding rate on the hydrophobic collector *v*_*Hy*_, and on the hydrophilic collector *v*_*max*_.

#### Particle counting of PET using light extinction (particles > 1 µm) as a processing control to*ol*

A suspension of aged PET was prepared by homogenizing 2.81 g of powder in 100 ml in Type-1 water. The mixture was sonicated and stirred for 15 min to re-suspend the particles. The PET suspension was then allowed to settle for 45 min. A 50 ml supernatant fraction was measured using a PAMAS particle counter (PAMAS, SVSS, Rutesheim, DE) following a dilution 1000 times with Type-1 water. Due to the density difference between PET (1.4 g/cm^3^) and water (1.0 g/cm^3^), the PET particles remaining in the supernatant are skewed towards smaller size than the original particle size distribution in the bulk.

#### Particle counting of PE using light extinction (particles > 1 µm) as a processing control tool

A suspension of 0.2355 g of dried aged LDPE was prepared in 50 ml ethanol (VWR, GPR Rectapur, Ethanol 99% (V/V)) by sonication and stirring (15 min). The difference in density between ethanol (0.789 g/cm^3^) and LDPE (0.910–0.940 g/cm^3^) was exploited. Smaller particles tend to stay longer in suspension, while larger particles have higher sedimentation rates just as described for PET above. After 45 min, the supernatant was analyzed using the PAMAS particle counter; the sample was diluted 1000 times, e.g., 0.1 ml in 100 ml Type-1 water.

#### Particle size distribution of the pristine cryo-milled PET and PE particles by laser diffraction measurements

The particle size distributions of the cryomilled powders were measured using a Sympatec-Helos laser diffraction instrument (Sympatec, Clausthal-Zellerfeld, DE). The powders were gradually added to a 50-ml cuvette with 2-propanol under constant stirring until an optical concentration of 20 % was achieved. The laser diffraction system employed two lenses that provided a measurement range from 0.5 to 875 µm. The equivalent sphere diameters were obtained from the diffraction patterns using the Fraunhofer approximation. Each MP powder was measured in triplicate.

#### Particle counting of the PET material using a single-particle extinction and scattering (SPES) method (> 0.5 µm)—for particle number concentration measurements

A Classizer™One (EOS S.r.l., Milan, IT. Software version ClassizerONE S1.4.34) was used to determine the number concentration of particles from 0.5 to approximately 5 µm. First, 1.4 g of dried, aged PET was suspended in 50 ml Type-1 water by sonication and stirring (15 min). The suspension was pulse filtrated over a 5 µm syringe inline filter (PALL, Acrodisc 32 mm Syringe Filter, 5 µm Supor Membrane). The < 5 µm PET suspension was diluted 40 time in Type-1 water and analyzed with the Classizer.

#### Particle counting of the PE material using a single-particle extinction and scattering (SPES) method (> 0.5 µm)—for particle number concentration measurements

A filter-cake of PE from the preparation process was re-suspended in 50 ml Type-1 water by sonication and stirring (15 min). The suspension was then pulse filtrated over a 5 µm syringe inline filter (PALL, Acrodisc 32 mm Syringe Filter, 5 µm Supor Membrane). The aged LDPE suspension with particle size < 5 µm was diluted 20 times in Type-1 water and analyzed with the Classizer.

#### FTIR microscopy of aged vs. pristine PET

PET particles were collected on a polytetrafluoroethylene (PTFE) filter (pore size 10.0 µm, Omnipore Membrane Filter, Merck) by filtration and drying. The PTFE filters were then analyzed by FTIR microscopy Nicolet iN10 FTIR Microscope, Thermo Fisher Scientific (Madison, WI, USA). The entire surface of the filter was scanned and the spectrum of each particle was collected (100 µm step-size scanning, 150 × 150 µm aperture, spectral resolution 16 cm^−1^, reflectance mode, spectral range 1300 to 4000 cm^−1^). The obtained spectra were identified based on their correlation with known spectra in the reference libraries (Pearson correlation, threshold match 75%, in-house and commercial spectral library).

For PET, a second FTIR microscope was also used. Analysis of the PET particles was performed after collecting them on an Anodisc filter (25 mm diameter, 100 nm pore size). The PET suspension was transferred to the Anodisk filter by depositing drops with a pipette at the same location allowing it to dry. The process was repeated until the deposited layer became thick enough to gain good-quality FTIR spectra. Measurements were done in transmission mode using a Bruker Hyperion 3000 FTIR microscope equipped with a 15 × objective and focal plan array (FPA) detector employing 16 scans, 2 × 2 binning of the 64 × 64 focal plane array (about 6 µm resolution), 8 cm^−1^ spectral resolution. The collected hyperspectral images were analyzed using an independent software solution for MP recognition (Purency).

#### FTIR microscopy of aged vs. pristine PE

The particles were collected on a polytetrafluoroethylene (PTFE) filter as described above (pore size 10.0 µm, Omnipore Membrane Filter, Merck). The PTFE filters were then analyzed by FTIR microscopy, Nicolet iN10 FTIR Microscope; Thermo Fisher Scientific, (Madison, WI, USA). The entire surface of the filter was scanned and the spectrum of each pinpointed particle was determined (100 µm step-size scanning, 150 × 150 µm aperture, spectral resolution 16 cm^−1^, reflectance mode, spectral range 1300–4000 cm^−1^). The obtained spectra were identified based on their correlation with known spectra in the reference libraries (Pearson correlation, threshold match 75 %, in-house and commercial spectral library).

#### Raman microscopy of aged vs. pristine PET and PE

Two different instruments were used to obtain Raman spectra of the aged and pristine PET and PE particles. First, an alpha 300 Raman microscope (WITec, Ulm, DE) equipped with a 532 nm laser was used to analyze the particles to obtain Raman spectra. The PE and PET suspensions were diluted in ethanol (ratio 1:3) and then were spotted on a silicon wafer. After drying, the samples were measured by Raman microscopy. Spectra were collected using a 10 × objective typically using 1 s integration time by averaging 30 accumulations. Thereafter, an inVia™ Qontor® confocal Raman microscope (Renishaw, Wooton-under-Edge, UK) equipped with 785 nm laser and 20 × objective was operated at 10 % laser power and 1 s accumulation time averaging 5 accumulations in addition.

#### Initial investigation of PE reactivity in acidic media and measurements of potassium content in the modified PET and PE using ICP-MS

Low-density polyethylene (LDPE) can be fully digested through oxidation by placing it in a microwave digestion system in a mixture of 5 ml HNO_3_ (65 %) and 1 ml H_2_O_2_ (30 %). This method was adapted to oxidize the MP PE particles’ surface only by introducing polar groups on the surface and making the particles more hydrophilic. Therefore, a single reaction chamber microwave digestion system/UltraWAVE from Milestone (Sorisole, IT) was used to investigate partial digestion of PE surface and define gentle oxidizing “ageing” parameters. The temperature was recorded where the pressure in the microwave system was starting to increase which was an indication of initial digestion of the surface. The actual ageing process of PE was then performed in a beaker in an open system operating at that same temperature (70 °C).

The content of K in the PET and PE before and after chemical treatment was analyzed by inductively coupled plasma mass spectrometry (ICP-MS) following complete digestion using the same microwave digestion system (Ultrawave, Milestone). For PET, 0.5 g sample and 5 ml 65 % HNO_3_ (m/v) were added to the digestion vessel employing a ramp for 80 min to 180 °C and then ramp 20 min to 250 °C with a 30 min hold at 250 °C. Digestion was found to be incomplete and another 2 ml of HNO_3_ was added (after cooling) and the digestion was repeated obtaining a clear solution after the second digestion cycle. The digest was diluted to 25 ml and then again 1/5 in Type-1 water and measured on an ICP-MS iCAP TQ from Thermo Scientific (Dilbeek, BE). The stable isotope ^39^ K was measured in Single Quad mode with kinetic energy dispersion, whereby 12 ml/min He was added to the collision cell. As an internal standard, ^89^Y was used.

Measurements of K-content in the modified PE were performed on 0.2 g samples in a similar fashion except for the addition of 0.5 ml H_2_O_2_ (30 %) and use of one digestion cycle.

#### Analytical quality control of the analytical methods and measurement techniques employed

The laser diffraction measurements for the particle size distribution were performed using a method that is under an ISO/IEC 17025 accreditation scope at JRC Geel. Similarly, the measurements of K-content in PET and PE were performed in a JRC laboratory that is accredited according to ISO/IEC 17025. These specific measurements of potassium were however outside the accreditation scope. To assess the accuracy, a QC standard of 50 µg/kg K was prepared separately and was measured before and after the PET samples which resulted in a recovery of 94.2% and 97.1%, respectively. For PE, the QC standard was 20 µg/kg and it was measured before and after the PE samples which resulted in a recovery of 109.8% and 95.2%, respectively. The JRC laboratory that performed the Hy-index measurements participated in and coordinated an Inter-Laboratory Comparison, ILC. Four different nanomaterials were analyzed with respect to their hydrophobicity index by four to nine independent laboratories as described in the “Experimental” section and in reference 27. The *z*-scores of the JRC laboratory were all below 1, showing good agreement with the other laboratories’ results. The zeta potential and single-particle extinction and scattering measurements were performed using standard operating procedures using check samples. The Raman and FTIR microscopes were employed using internal and external spectral libraries. Samples were prepared in clean benches to avoid contamination when placing the samples onto the substrates.

## Results and discussion

### Stability of the artificially aged MP particles

**Fig. 1:**
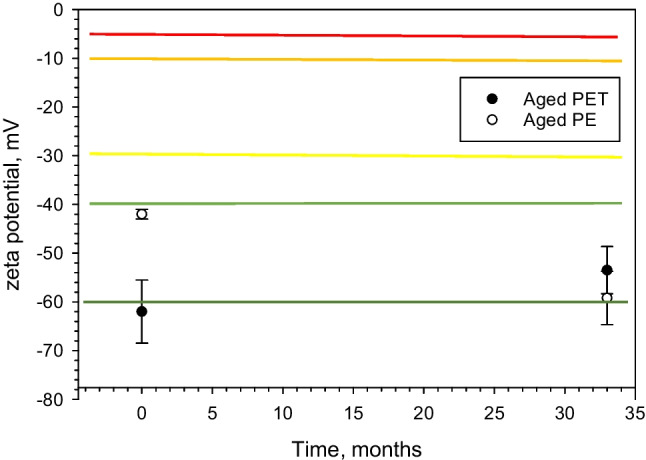
Zeta potential of aged PET and PE as a function of time after storage in dry powder form at room temperature. The respective colored lines indicate different zones of stability of colloidal suspensions where 0 to ± 5 mV results in rapid coagulation or flocculation, ± 10 to ± 30 mV indicates incipient instability, ± 30 to ± 40 mV shows moderate stability, ± 40 to ± 60 mV good stability, and > 61 mV excellent stability [33]

Access to artificially aged MP particles with a high degree of equivalence when subsampled from a suspension would increase the relevance and comparability of toxicological effect studies. This calls in principle for the production of reference materials of MP particles where the degree of hydrophilicity (i.e., the artificial age of MP) is reproducible and where this qualitative property can be confirmed using a quantitative method. The qualitative property which can be assigned is of ordinal nature, e.g., “less aged,” “aged,” and “more aged.” Zeta potential was used to evaluate the stability of suspensions prepared from the artificially aged MP as a function of time. The result is shown in Fig. 1 where the zeta potential of PET and PE suspensions was measured with three to six replicates at the time of preparation and after storage at room temperature as dry powders for 33 months. All measurement results essentially fall within the “good stability region” between − 40 and − 60 mV [33]. It can indeed be seen in Fig. 1 that the observed change in the quantitative zeta potential values over time does not affect the hydrophilicity (used as a proxy for the artificial age) as these materials remained in the good stability region for their respective suspensions [32, 33]. Based on these measurements, we conclude that the artificially aged MP particles in dry powder form remain hydrophilic after a storage time of almost 3 years. The 33 months are coincidentally similar to the age of MP found in the North Pacific Subtropical Gyre reported by Brandon et al. [2].

### Hydrophobicity index, Hy

**Fig. 2:**
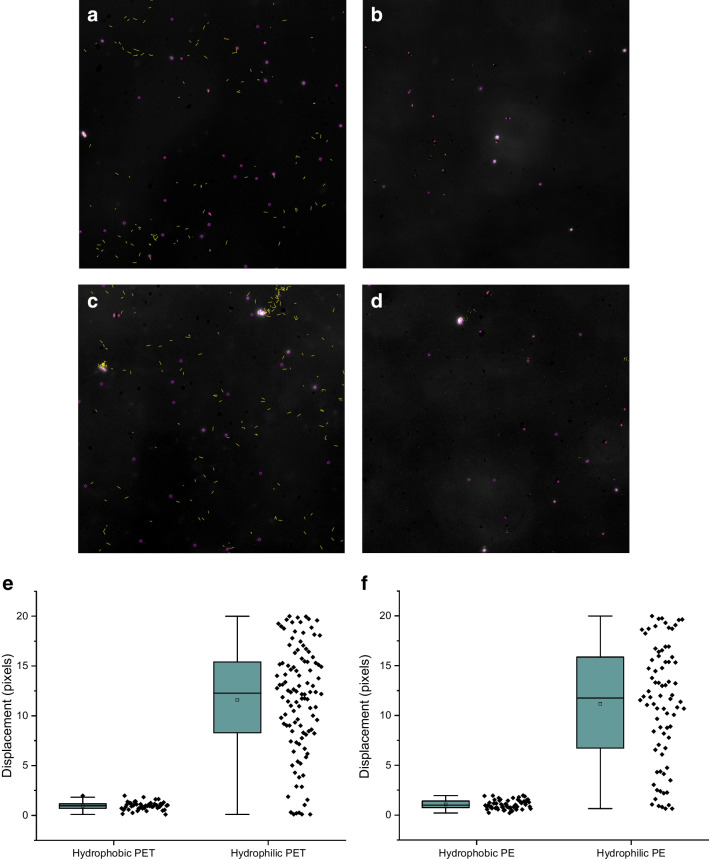
**a** Dark field microscopy image of artificially aged PET (first frame). The purple dots show the identified particles in the initial frame of the recorded sequence (particles in focus with the surface). The yellow lines illustrate the displacement of artificially aged polyethylene terephthalate (PET) particles in contact with a hydrophobic collector over a 12-min period. (The higher number of tracks compared to the particle count is due to the display of all movements across 25 frames.) Hydrophilic PET particles exhibit a lower affinity for the hydrophobic collector, as denoted by their higher displacement values (e.g., longer tracks). The hydrophilic PET particles have a lower adhesion tendency. **b** Dark-field microscopy image of pristine PET (first frame). The purple dots show the identified particles in the initial frame of the recorded sequence (particles in focus with the surface). A lower displacement value (number of pixels covered) suggests a stronger affinity of pristine hydrophobic PET particles for the hydrophobic collector (e.g., shorter yellow tracks). This implies that hydrophobic pristine PET particles tend to adhere more strongly to the hydrophobic collector. **c** Dark-field microscopy image of artificially aged PE (first frame). The purple dots show the identified particles in the initial frame of the recorded sequence (particles in focus with the surface). The yellow lines illustrate the displacement of artificially aged polyethylene (PE) particles in contact with a hydrophobic collector over a 12-min period. (The higher number of tracks compared to the particle count is due to the display of all movements across 25 frames.) Hydrophilic PE particles exhibit a lower affinity for the hydrophobic collector, as denoted by their higher displacement values (e.g., longer tracks). The hydrophilic PE particles have a lower adhesion tendency. **d** Dark-field microscopy image of pristine PE (first frame). The purple dots show the identified particles in the initial frame of the recorded sequence (particles in focus with the surface). A lower displacement value (number of pixels covered) suggests a stronger affinity of pristine hydrophobic PE particles for the hydrophobic collector (e.g., shorter yellow tracks). This implies that hydrophobic pristine PE particles tend to adhere more strongly to the hydrophobic collector. **e** Box and whisker plots showing the displacements of 23 hydrophobic particles and 34 hydrophilic particles of PET over 25 sequential DF images. A total of 47 hydrophobic and 110 hydrophilic tracks were identified. The mean value for the displacement of hydrophobic particles is 0.95 ± 0.40 pixels, while for hydrophilic particles, it is 11.59 ± 5.42 pixels. This shows that hydrophilic particles exhibit much larger displacement rates than hydrophobic particles. **f** Box and whisker plots showing the displacements of 27 hydrophobic particles and 32 hydrophilic particles of PE over 25 sequential DF images. A total of 56 (for the hydrophobic PE) and 78 (for the hydrophilic PE) tracks were identified. The average displacement for hydrophobic particles is 1.06 ± 0.45 pixels, while for hydrophilic particles, it is 11.15 ± 5.88 pixels. This indicates that hydrophilic particles exhibit much larger displacement rates than hydrophobic ones

The Hy-index is the logarithm of *v*_*Hy*_*/v*_*max*_ where *v*_*Hy*_ is the slope of the binding rate to the hydrophobic collector surface and *v*_*max*_ is the slope of the maximum binding rate due to attractive electrostatic interactions. Practically, Hy-index is a direct measurement of the affinity of a substance, such as artificially aged MP, to bind to a hydrophobic collector rather than remaining in the aqueous phase. The average Hy-index value for artificially aged PET was − 1.61 ± 0.21 (*n* = 9). Whereas for the artificially aged PE, a Hy-index value of − 1.20 ± 0.05 (*n* = 9) was found. The corresponding Hy-index values for pristine PET and pristine PE were − 0.124 ± 0.06 (*n* = 9) and − 0.343 ± 0.113 (*n* = 9), respectively. According to the OECD guideline 126, materials with Hy-index values below − 1 are considered hydrophilic [26]. The closer the Hy-index value is to 0, the more hydrophobic the material is. Briefly, a Hy-index value of − 1 indicates that only 10% of the particles adhere to the hydrophobic surface used in the experiment because the Hy-scale follows a logarithmic progression [26]. For comparison, the most hydrophilic material measured to date is SiO_2_ (Hy = − 2.5) [26], and the most hydrophobic was milled pristine PET particles as reported here (Hy = − 0.124). A gradual decrease of the surfactant concentration would provide gradually decreasing Hy-index values for pristine MP particles. In this fashion, a backwards extrapolation to the Hy value that represents zero surfactant concentration can be done [26]. However, in this study, we used the lowest possible surfactant concentration needed for stable particle suspension. Therefore, the pristine MP particles of PET and PE were measured in the presence of 0.001% surfactant (Triton X100). In Fig. 2a and b, the respective displacements of artificially aged and pristine PET particles are shown (DF microscopy images) where the corresponding displacements for artificially aged and pristine PE are shown in Fig. 2c and d. The displacement recorded by DF microscopy expressed as number of pixels covered during 12 min is associated with the binding rates to the hydrophobic substrate. In Fig. 2e and f, box and whisker plots are shown for the number of pixels travelled in the DF images by the respective hydrophilic and hydrophobic particles. In both cases, it can clearly be seen that the pristine hydrophobic MP particles have a much higher affinity to the hydrophobic substrate.

**Fig. 3:**
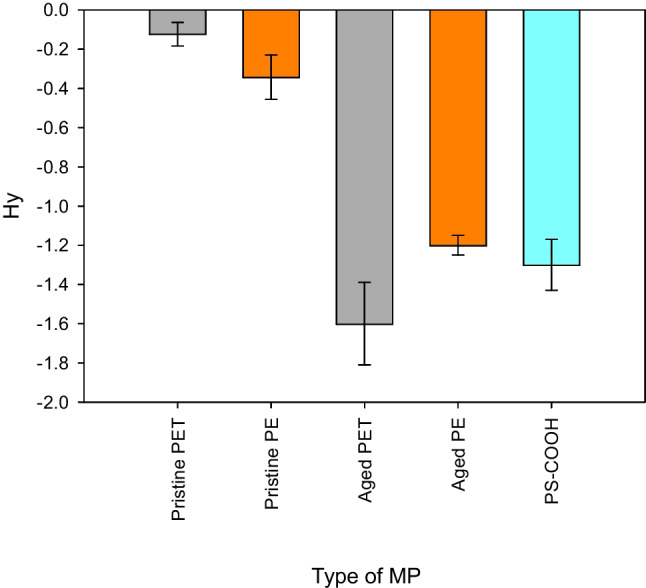
Hy-index of different MP fragments following filtration over a cellulose acetate filter with a < 1.2 µm cut-off. The pristine MP of PET and PE has very low Hy-index values in contrast to the artificially aged PET and PE. The error bars are relative standard deviations for (*n* = 9). PET is depicted in grey, PE in orange, and PS in light blue, respectively

Other materials, such as carboxylate-functionalized polystyrene beads (PS), have a Hy of approximately − 1.3 ± 0.13 (*n* = 9) [26]. The Hy-index values for the artificially aged PET and PE were thus found to be similar to that of carboxylate-functionalized polystyrene. The similarity in Hy-index values between artificially aged PET and PE particles and PS beads with COOH groups is consistent with the expectation that these materials exhibit hydrophilic properties. The Hy-index values for the different polymers (in the form of aged and pristine MP fragments) are illustrated in Fig. 3. Furthermore, the zeta potential results align with the Hy-index results, where the artificially aged PE exhibits a less negative zeta potential (− 42 mV) compared to PET (− 62 mV). This difference in zeta potential values also supports the conclusion that the artificially aged PET is probably more hydrophilic than the artificially aged PE.

The Hy-index measurements offer the possibility to compare two values of the hydrophilicity of different or the same MP particles which are of relative rank order. This could be the basis for an ordinal property value for qualitative RMs of artificially aged MP where the certified value is based on Hy-index measurements. The methodology of measuring Hy-index using dark-field microscopy appears to be mature enough based on the results from a recent inter-laboratory comparison with nine independent participants and the adoption of a test-guideline (TG) by the OECD [26, 27]. Therefore, it should be feasible to certify Hy-index values based on data from inter-laboratory comparison studies. Another example of relative rank order values is Mohs hardness scale for minerals which is an ordinal scale for a qualitative property beginning with talc at 1 to diamond at 10 as explained in ISO 33406 [24].

### Uniformity of the particle number concentration of artificially aged MP particles for reproducible spiking

**Fig. 4:**
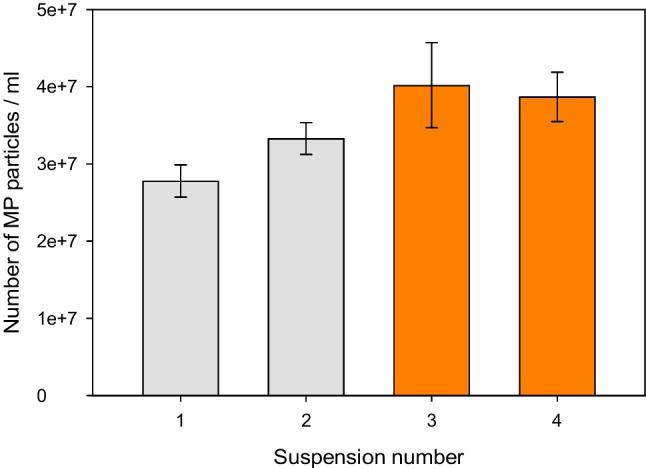
Uniformity of the particle number concentration per ml when spiking aged MP particles measured by single-particle extinction and scattering (SPES). Each bar shows the particle number/milliliter for a specific suspension where the grey bars show PET suspensions and the orange bars show PE suspensions. The suspensions were prepared from two different batches of artificially aged MP particles per polymer. The error bars show ± one standard deviation for *n* = 6 replicates. Each suspension was measured twice with three replicates within a ten-day period

In previous work, we described the versatility of suspensions when working with particles for a whole range of different applications, especially for the preparation of new kinds of reference materials [36]. Also in this work, the ultimate use of these artificially aged MP particles is to prepare suspensions and spike the MP in a controlled manner into different matrices for toxicological effect studies or for other purposes. The possibility of spiking a specific number of particles from an MP suspension must have a high enough precision to improve the statistical significance between different effect studies. The spiking entails a degree of equivalence with respect to the number of MP particles added per experiment where the degree of hydrophilicity must remain unchanged. In Fig. 4, the standard deviations of the particle number per milliliter are shown for four different suspensions. The suspensions were filtered through a 5 µm inline syringe filter (the majority of the particles were nevertheless below 1 µm). For practical use of the aged MP particles, it is recommended to use freshly prepared suspensions that are thoroughly mixed which have been assessed with respect to their particle number per milliliter to be sure about the actual spiking level. A test with respect to the stability of the suspensions measured within a 10-day period provided the following data. For freshly prepared suspensions, the relative standard deviations (RSDs) of the particle count/ml ranged from 1.8 to 8.7%. After 10 days in suspension, the overall RSDs increased from 6.2 to 13.7%. Figure 4 clearly shows that reproducible spiking of artificially aged MP particles is possible and the suspensions can be used repeatedly for at least 10 days.

### Results of particle size and number distribution measurements obtained by laser diffraction

The particle size distribution was measured for the cryo-milled PET and PE particles in their pristine state before chemical treatment. Equivalent sphere diameters (ESD) were obtained in triplicate for 36 different size classes from 0.5 to 875 µm. For PET, the average ESD for X_10_, X_50_, and X_90_ of the cumulative volume distribution was 21.6, 83.2, and 206.8 µm, respectively. For PE, the corresponding average ESDs were for X_10_, X_50_, and X_90_ 16.6, 78.3, and 276.3 µm, respectively. The corresponding cumulative number distribution showed that 95% of the particles for both PET and PE were below 2.5 µm. This particle size distribution is similar to the BAM-P201 reference material with the exception that particles below 5 µm in size, were more abundant in our materials than visible in the PSA data for the BAM-P201 material [11]. If the BAM material would be milled further, pristine surfaces would be exposed and the resulting particles would be more hydrophobic and less relevant for toxicological effect studies.

### Results using spectroscopic techniques—FTIR microscopy of PET and PE

No major differences in the spectra between aged and pristine MP could be observed using FTIR microscopy. Both pristine and aged PET were recognized as PET when matching with the spectral libraries. Similarly, there was no major difference between the spectra of aged and pristine PE. The spectral matching data is shown in Table 1 (Nicolet microscopy data). Likewise, identification of the aged MP particles of PET using the Bruker Hyperion FTIR microscope resulted in correct identification in comparison with pristine PET (the aged PE was not measured using the Bruker FTIR microscope). The conclusion must be that the FTIR microscopy is not sensitive enough to pick up any major differences between aged and pristine MP that have occurred on the surface since transmission measurements were performed. The good spectral matching for the aged PE and PET particles still makes it possible to detect them in different samples when applying this commonly used technique in the area of MP measurements. More work would be needed to optimize measurements that possibly could reveal subtle differences between aged and pristine MP.

**Table 1:** Identification of pristine and artificially aged MP using a Nicolet iN10 FTIR microscope. The obtained spectra were identified based on their correlation with known spectra in the reference libraries (Pearson correlation, threshold match 75%, compared with an in-house and a commercial spectral library)

Polymer analyzed	Identified as:	Matching (%)
Alfa Aesar PE starting material	LDPE	99
Artificially aged PE	PE	98
Goodfellow PET starting material	PET	98
Artificially aged PET	PET	97

### Results using spectroscopic techniques—Raman microscopy of PET and PE

In this work, two different Raman instruments were employed for identification of the MP particles using built-in and online spectral libraries. In addition, the spectra of pristine and aged particles were compared in order to evaluate any changes in peak shape, relative peak height, and position and interpret these changes with the resources available in literature. Firstly, Raman spectra were acquired using a WITec alpha 300 Raman microscope employing a 532 nm laser. The collected data were compared with the free online library Openspecy [37] and Pearson’s correlation match in the range of 0.99 to 0.97, showing no major difference between the pristine and the aged polymers for both PET and PE. To minimize the possible effect of the fluorescence that was detected in PET and PE particles with the 532 nm wavelength laser, Raman spectra were also collected using 785 nm with a Renishaw instrument. In this case, the spectral comparisons were performed with the instrument’s internal spectral library. At first glance, no major differences were found between pristine and the aged polymers for both PET and PE either. The overall spectral matching data obtained by both instruments are shown in Tables 2 and 3 (Witec and Renishaw data, respectively). The full Raman spectra of PET and PE are shown in Figures S3 to S6. Nevertheless, when performing in-depth comparisons, slight differences between aged and pristine MP particles were revealed as detailed below.

**Table 2:** Identification of pristine and artificially aged MP using an alpha 300 Raman microscope from WITec. The obtained spectra were identified based on their correlation with known spectra from the Openspecy on-line library [37]

Polymer analyzed	Identified as:	Matching (%)
Alfa Aesar PE starting material	LDPE	99
Artificially aged PE	PE	98
Goodfellow PET starting material	PET	98
Artificially aged PET	PET	97

**Table 3:** Identification of pristine and artificially aged MP using the Renishaw Raman microscope. The obtained spectra were identified based on their correlation with the built-in spectral library

Polymer analyzed	Identified as:	Matching (%)
Alfa Aesar PE starting material	PE	99
Artificially aged PE	PE	99
Goodfellow PET starting material	PET	99
Artificially aged PET	PET	99

For the additional comparisons, background-subtracted spectra of pristine and aged MP particles were normalized to a reference peak at 1615 cm^−1^ for PET and 1296 cm^−1^ for PE [38, 39]. In the case of PET MP, the reference peak corresponds to a C = C aromatic stretching.

The spectra of pristine and aged PET MPs are still mostly overlapping. A slight difference was observed for the peak ratio of the 1095 and 1120 cm^−1^ peaks, which is correlated with a structural change in the crystalline/amorphous phase during the chemical ageing process [40]. These spectra are shown in Figures S7 and S8, respectively.

The PE spectra were normalized to the CH_2_ twist at 1296 cm^−1^, which is known to remain consistent regardless of polymerization or degradation. According to the literature, the three main Raman spectral regions for pristine LDPE are as follows: region I, 1040–1200 cm^−1^, which is dominated by C–C stretching; region II, around 1300 cm^−1^, consisting of –CH_2_- twisting; and region III, 1350–1500 cm^−1^ dominated by –CH_2_- bending [39]. Analysis of the three different areas in the spectra shown in Figures S9 and S10 highlight that aged PE exhibits a small but clear difference in terms of intensity and wavelength shifts with respect to pristine PE. This effect was as also observed by Phan et al. [39]. These results suggest that other structural changes are occurring during the chemical treatment process that not only result in surface modifications as observed using other techniques such as Hy-index and the high amount of incorporated potassium (see below) [41]. Significant oxidation has most likely occurred on the surface of the artificially aged MP particles but Raman microscopy is not sensitive enough to show consistent and measurable variation in spectral features associated with these surface modifications. Accordingly, further investigations are needed with dedicated techniques, for instance by using X-ray photoelectron spectroscopy (XPS) that is more sensitive to changes on the particle surface.

### Measurement of potassium on the surface of PET and PE using ICP-MS

The amount of potassium in the pristine PET (before ageing) was below the level of detection. After treatment, it increased to 18.05 and 14.87 mg/kg respectively in two samples of aged PET from two different batches. These results indicate the presence of COO^−^K^+^ polar groups on the surface of the PET particles. With respect to PE, the amount of potassium in the pristine PE (before ageing) was also below the level of detection. After treatment, it increased to 4.7 and 4.2 mg/kg respectively in two samples of aged PE (from two different batches). This result also indicates the presence of COO^−^K^+^ polar groups on the surface of the PE particles. Hence, the good correlation for the Hy-index for PS beads with COOH groups is not surprising. Indeed, the higher zeta potential for aged PET in comparison with aged PE is directly correlated with the higher potassium content for PET and the higher Hy-index as well.

## Conclusions

By applying cryogenic milling and chemical treatments with base and acid, PET and PE particles were aged artificially, making them hydrophilic. This eliminates the need for surfactants when suspending aged PET and PE particles in water when used for toxicological effect studies. Milling larger amounts of pristine polymers also results in larger quantities of artificially aged MP in comparison with approaches involving ultrasonication or direct UV treatment of larger fragments only. We see no practical limitations to modify kg-amounts of cryogenically milled MP particles in order to make them hydrophilic. The described method of ageing MP results in dry powders that can be converted to aqueous suspensions that are stable without the addition of surfactants. Tests suggest that suspensions can be reproducibly sampled for about 10 days after their preparation. The MP particles can in addition be added to sediments or biota with a much reduced risk for segregation between sediment particles, biota, and the added MP. FTIR and Raman spectra show that the aged MP particles are still intact enough to be recognized as the respective polymers when using FTIR microscopy and Raman microscopy. Small differences in Raman Spectra were nevertheless revealed between aged and pristine PET and PE following a more thorough spectral analysis. The most important result and outlook is that the hydrophilic, hence artificially aged MP particles, could in the future become qualitative reference materials. They should be characterized for their identity, a nominal property, and an ordinal property value reflecting their artificially aged status based on Hy-index measurements.

## Supplementary Information

Below is the link to the electronic supplementary material.

Supplementary file1 (DOCX 2417 KB)
