# Supplementary material for: Approaches for the preparation and evaluation of hydrophilic polyethylene and polyethylene terephthalate microplastic particles suited for toxicological effect studies
Source: Anal Bioanal Chem. 2025 Jan 25;417(12):2589–602. doi: 10.1007/s00216-024-05726-7 (PMC12003597; doi:10.1007/s00216-024-05726-7)

SUPPLEMENTARY MATERIAL

**Figure S1.** Schematic overview of the preparation of artificially aged PET.

**Figure S2.** Schematic overview of the preparation of artificially aged PE.

**Figure S3.** Raman spectra of pristine PET and artificially aged PET using the WITec Raman microscope applying 532 nm excitation.


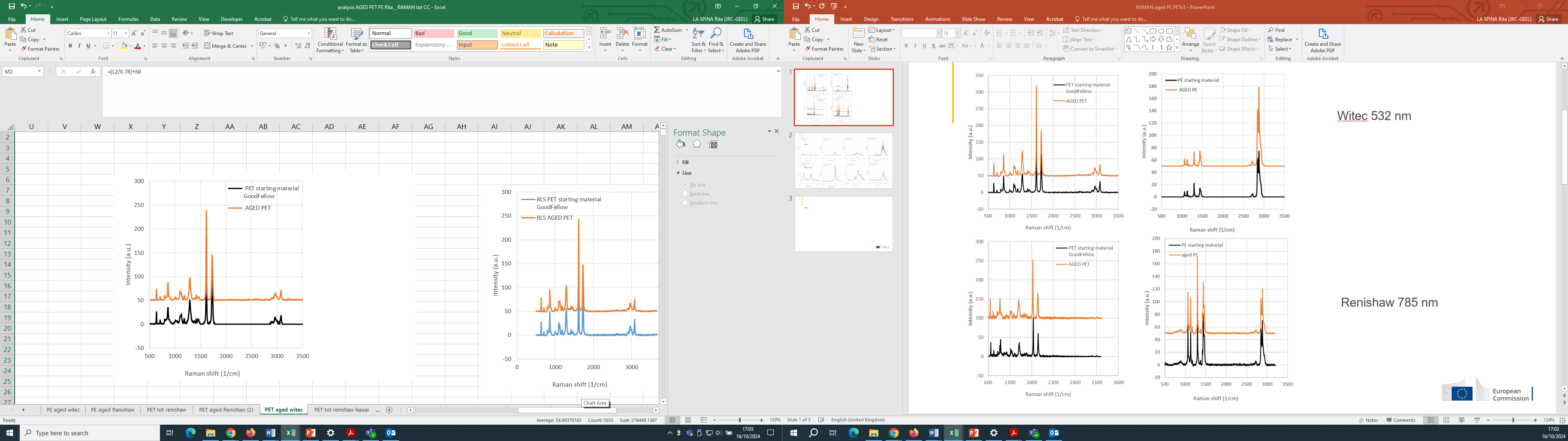


**Figure S4.** Raman spectra of pristine PE and artificially aged PE using the WITec Raman microscope applying 532 nm excitation.


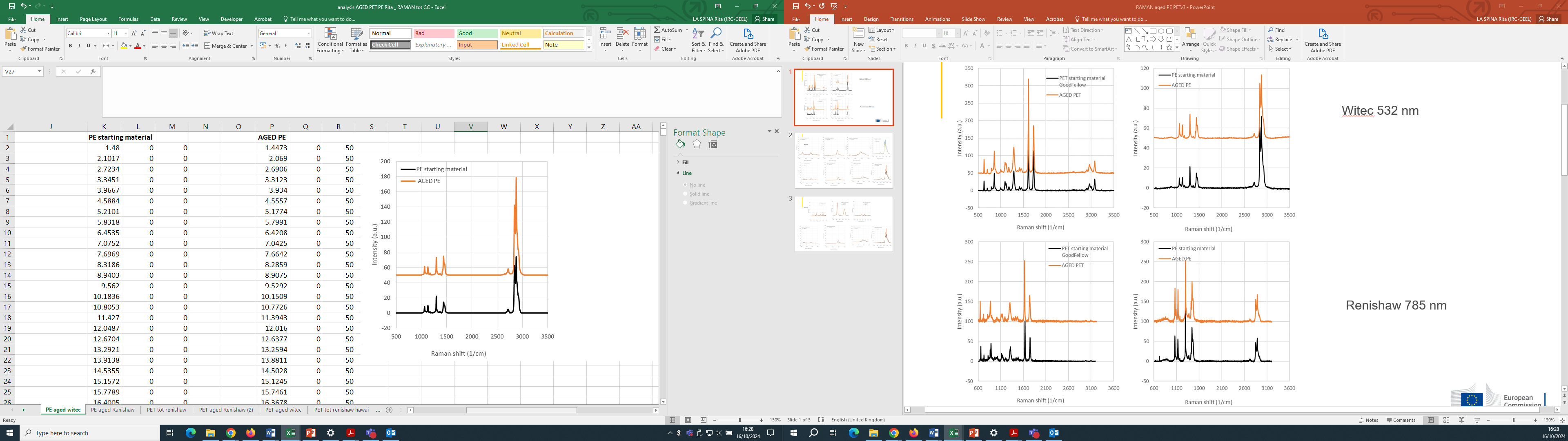


**Figure S5.** Raman spectra of pristine PET and artificially aged PET using the Renishaw Raman microscope applying 785 nm excitation.


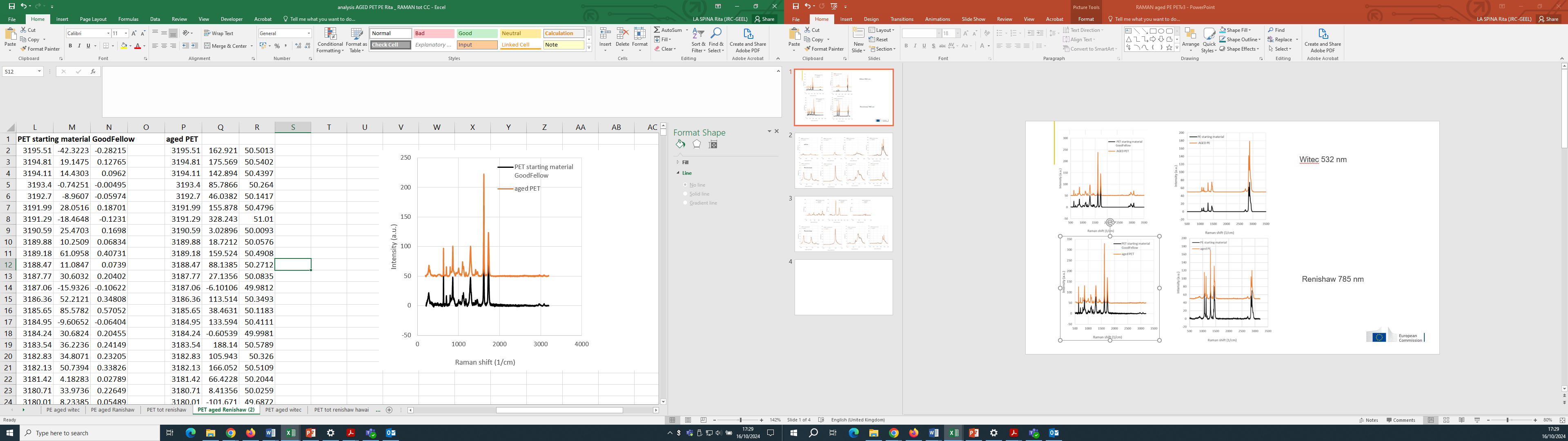


**Figure S6.** Raman spectra of pristine PE and artificially aged PE using the Renishaw Raman microscope applying 785 nm excitation.


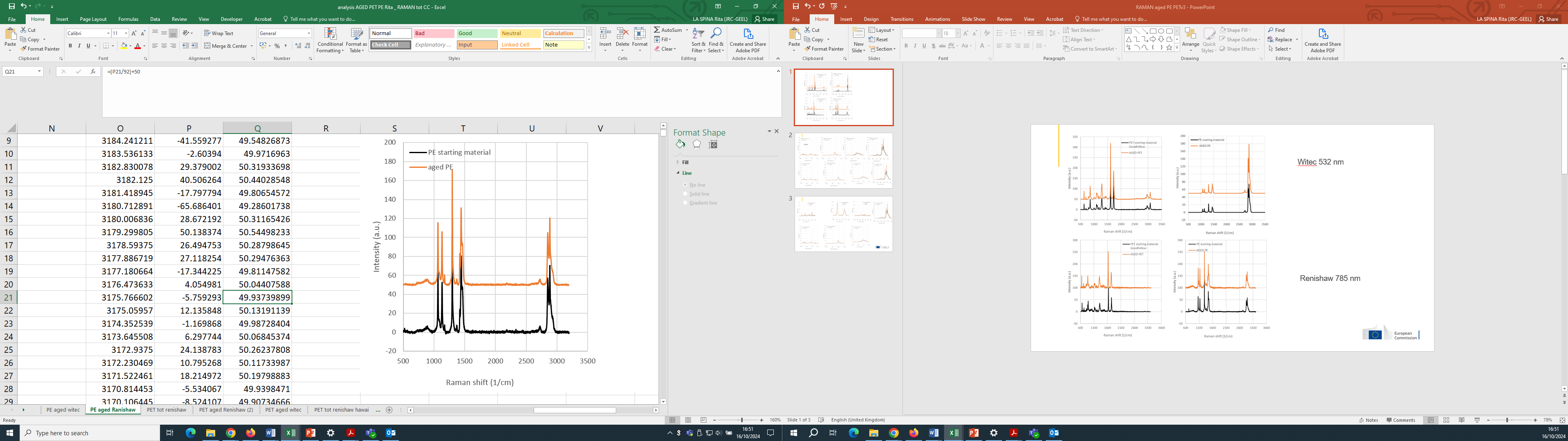


**Figure S7.** Raman spectra of pristine PET and artificially aged PET using the WITec Raman microscope applying 532 nm excitation. To the right, the reference peak at 1615 cm^-1^ used for normalisation is shown. To the left, slight differences for the peaks at 1095 and 1120 cm^-1^ can be observed.


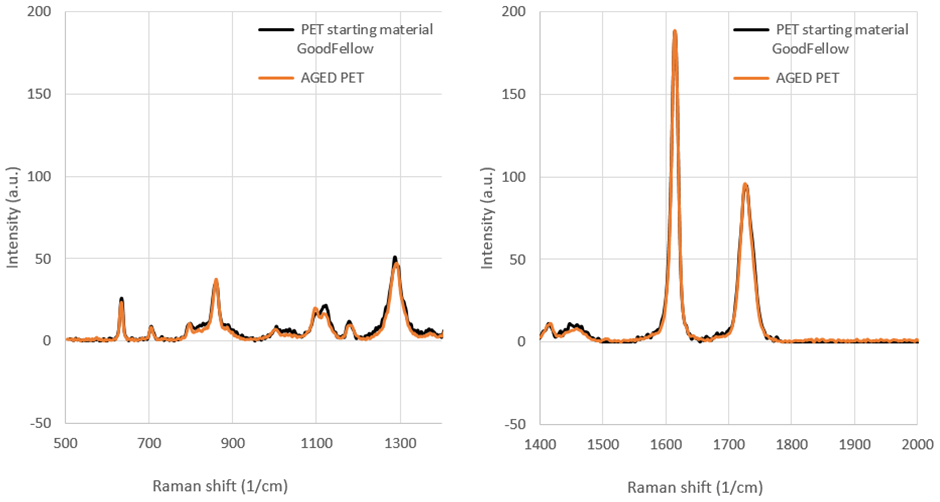


**Figure S8.** Raman spectra of pristine PET and artificially aged PET using the Renishaw Raman microscope applying 785 nm excitation To the right, the reference peak at 1615 cm^-1^ used for normalisation is shown. To the left, slight differences for the peaks at 1095 and 1120 cm^-1^ can be observed.


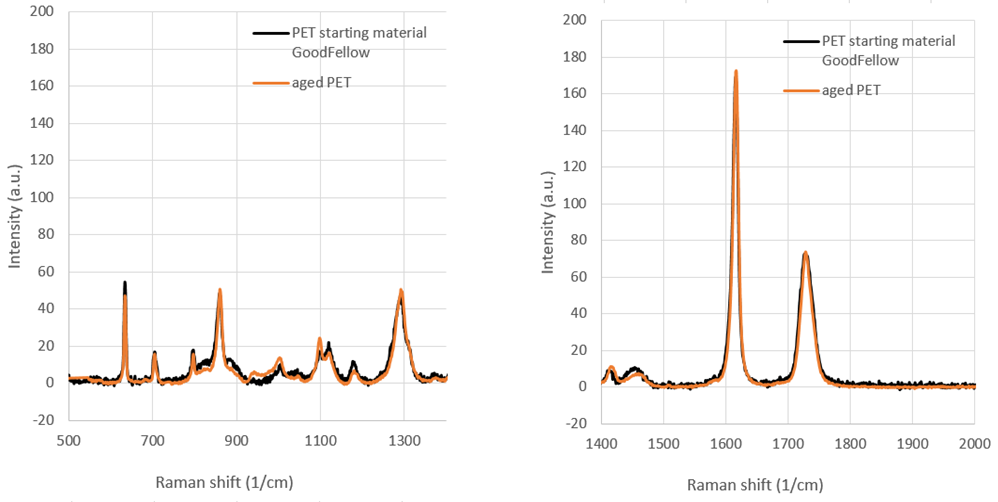


**Figure S9.** Raman spectra of pristine PE and artificially aged PE using the WITec Raman microscope applying 532 nm excitation. The three spectra correspond to the three analysed regions discussed in the main text: Region I to the left from 1040 to 1200 cm^-1^, Region II in the middle, around 1300 cm^-1^, (peak used for normalisation) and Region III to the right from 1350 to 1500 cm^-1^.


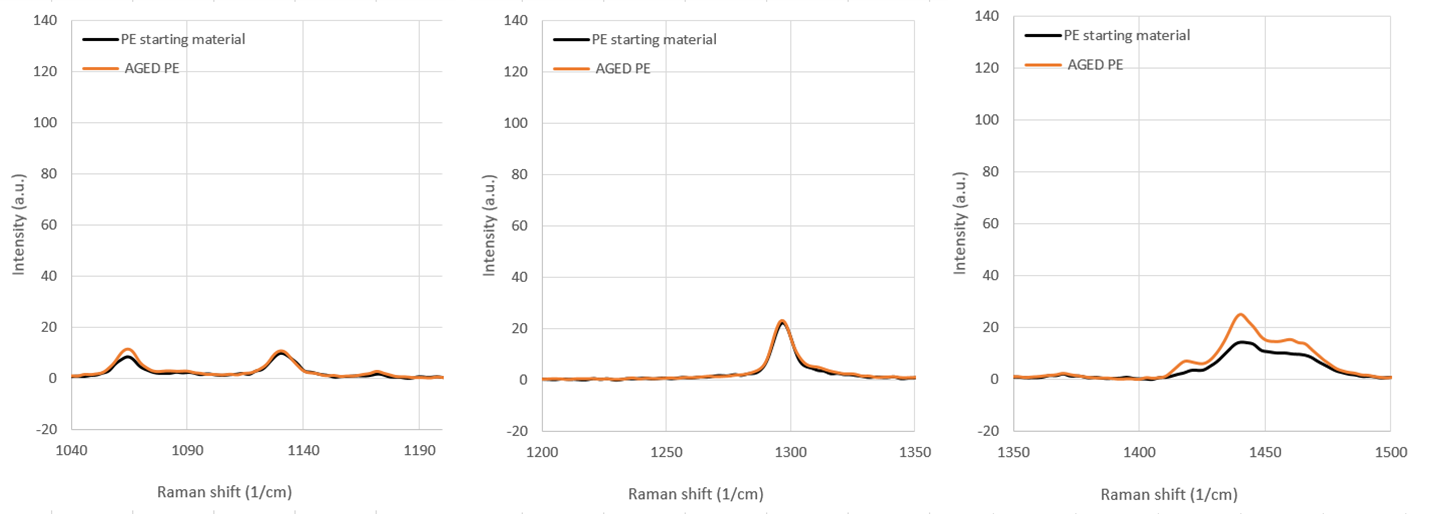


**Figure S10.** Raman spectra of pristine PE and artificially aged PE using the Renishaw Raman microscope applying 785 nm excitation. The three spectra correspond to the three analysed regions discussed in the main text: Region I to the left from 1040 to 1200 cm^-1^, Region II in the middle, around 1300 cm^-1^, (peak used for normalisation) and Region III to the right from 1350 to 1500 cm^-1^.


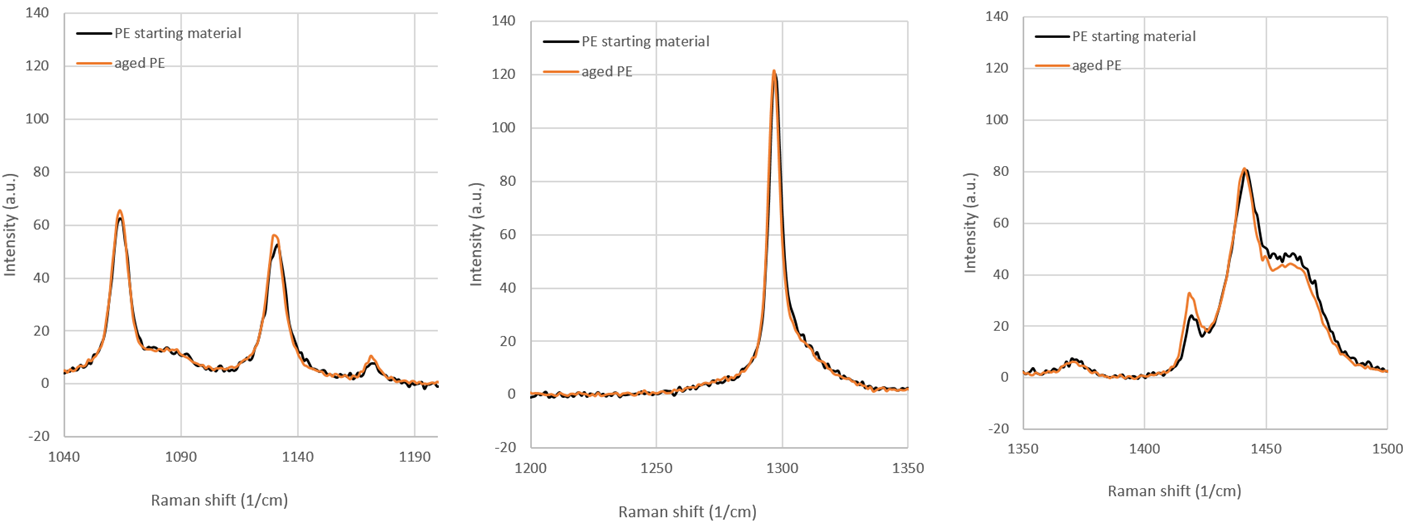

Supplement: Supplementary file 1 — Supplementary file1 (DOCX 2417 KB) [file 216_2024_5726_MOESM1_ESM.docx]
